# The Forkhead Box Gene, *MaSep1*, Negatively Regulates UV- and Thermo-Tolerances and Is Required for Microcycle Conidiation in *Metarhizium acridum*

**DOI:** 10.3390/jof10080544

**Published:** 2024-08-02

**Authors:** Tiantian Song, Chan Li, Kai Jin, Yuxian Xia

**Affiliations:** 1 Genetic Engineering Research Center, School of Life Sciences, Chongqing University, Chongqing 401331, China; 202226021001@cqu.edu.cn (T.S.); 202026021001@cqu.edu.cn (C.L.); 2 Chongqing Engineering Research Center for Fungal Insecticide, Chongqing 401331, China; 3 Key Laboratory of Gene Function and Regulation Technologies Under Chongqing Municipal Education Commission, Chongqing 401331, China; 4 National Engineering Research Center of Microbial Pesticides, Chongqing 401331, China

**Keywords:** entomopathogenic fungus, *Metarhizium acridum*, MaSep1, stress tolerances, conidiation pattern shift

## Abstract

Insect pathogenic fungi have shown great potential in agricultural pest control. Conidiation is crucial for the survival of filamentous fungi, and dispersal occurs through two methods: normal conidiation, where conidia differentiate from mycelium, and microcycle conidiation, which involves conidial budding. The conidiation process is related to cell separation. The forkhead box gene *Sep1* in *Schizosaccharomyces pombe* plays a crucial role in cell separation. Nevertheless, the function of Sep1 has not been clarified in filamentous fungi. Here, MaSep1, the homolog of Sep1 in *Metarhizium acridum*, was identified and subjected to functional analysis. The findings revealed that conidial germination of the *MaSep1*-deletion strain (Δ*MaSep1*) was accelerated and the time for 50% germination rate of conidial was shortened by 1 h, while the conidial production of Δ*MaSep1* was considerably reduced. The resistances to heat shock and UV-B irradiation of Δ*MaSep1* were enhanced, and the expression of some genes involved in DNA damage repair and heat shock response was significantly increased in Δ*MaSep1*. The disruption of *MaSep1* had no effect on the virulence of *M. acridum*. Interestingly, Δ*MaSep1* conducted the normal conidiation on the microcycle conidiation medium, SYA. Furthermore, 127 DEGs were identified by RNA-Seq between the wild-type and Δ*MaSep1* strains during microcycle conidiation, proving that MaSep1 mediated the conidiation pattern shift by governing some genes associated with conidiation, cell division, and cell wall formation.

## 1. Introduction

Insect pathogenic fungi have shown great potential in agricultural pest control [1,2]. *Metarhizium acridum* has been successfully applied as a mycoinsecticide to control locusts in Asia, Africa, and Australia [3,4]. Conidia are the infective unit and reproductive basis of entomopathogenic fungi, and are also the main active components of mycopesticides [5,6]. In general, conidia are mainly produced through solid-state fermentation, since they display more resistance to abiotic stress in the field [7]. However, substrate aggregation seriously affects production efficiency due to the rapid growth of hyphae during solid fermentation [8]. The two modes of asexual development are normal conidiation and microcycle conidiation in filamentous fungi [9]. Microcycle conidiation is the direct production of conidia by conidia, with almost no hyphae formation [10], which can make up for the lack of a solid-state fermentation process [11]. In addition, the quality of conidia produced by microcycle conidiation was obviously improved, with higher conidial production and higher resistance to abiotic stress, which are beneficial to the industrial production of entomopathogenic fungi [12]. Consequently, exploring the mechanism of conidiation pattern shift is of great significance in entomopathogenic fungi.

In specific circumstances, for instance, variations in nutrition or temperature, normal conidiation can be shifted to microcycle conidiation [13]. Some genes related to microcycle conidiation have been identified in *Metarhizium acridum*, such as *MaNCP1*, *MaCreA*, *MaMsn2*, *Mavib-1*, *MaSln1*, *MaSho1*, *MaPEPDA*, *MaPpt1*, *MaOpy2,* and *MaH1* [14,15,16,17,18,19,20,21,22]. In addition, *MaMsn2* and *MaNsdD* regulate microcycle conidiation by negatively regulating *MaAbaA*, in which *MaMsn2* is regulated by *MaH1* [23]. Nevertheless, the mechanisms that regulate conidiation pattern shift are still largely unknown.

The conidiation process is related to cell separation. The regulatory mechanism of cell separation was mainly focused on yeast [24]. In *Schizosaccharomyces pombe*, 16 cell separation-related genes Sep1-16 were identified. When these genes are deleted, the cells are unable to separate properly [25,26]. Sep1, as a transcription factor, belongs to the HNF-3/forkhead family, and *Sep1* deletion can hinder septum division [25]. The expression of genes involved in cell division is regulated by Sep1, Ace2, and Fkh2 [27]. Sep1 and Fkh2 share the same forkhead domain. These transcription factors are believed to play opposing roles in regulating transcription during mitosis [28]. The forkhead protein Sep1p triggers the ZnF protein Ace2p. The main role of the Sep1p-Ace2p transcription pathway is to activate gene expression necessary for completing cell separation and cytokinesis upon completion of the cell cycle [29]. The forkhead transcription factors contain a DNA-binding domain with a winged helix structure [30] and are conserved in fungi. The forkhead transcription factor Mo-HCM1, the homolog of Sep1 in *M. oryzae*, is important in fungal development [31]. CaFkh2p, the homolog of Sep1 in *C. albicans*, is crucial for morphogenesis and virulence [32]. FkhB, the homolog of Sep1 in *A. nidulans*, is essential for fungal growth, development, and stress tolerance [33]. However, the functions of Sep1 have not been clarified in insect pathogenic fungi.

In this research, MaSep1, the homolog of Sep1 in *M. acridum*, was identified and subjected to functional analysis. Disruption of *MaSep1* led to accelerated conidial germination, reduced conidial production, and increased resistance to heat shock and UV-B irradiation, with no impact on fungal virulence. Interestingly, the *MaSep1*-disruption strain could not perform microcycle conidiation on SYA medium. RNA-Seq analysis further revealed that MaSep1 regulates this conidiation pattern shift by influencing genes related to conidiation, cell separation, cell cycle, cell polarity, and cell wall formation.

## 2. Materials and Methods

### 2.1. Strains and Cultivations

The wild-type strain *M. acridum* CQMa102 (CGMCC 0877) and mutants derived from the wild-type strain were cultivated as described previously [14]. Routine DNA manipulation was conducted using *Escherichia coli* DH5α competent cells (TransGen Biotech, Beijing, China). Fungal transformations were carried out using *Agrobacterium tumefaciens* AGL-1 (Weidi Biotech, Shanghai, China). To observe conidiation processes, 100 μL conidial suspensions (1 × 10^7^ conidia/mL) from different fungal strains were evenly spread onto 1/4 SDAY and SYA media, and grown at 28 °C. Starting from 8 h, the conidiation patterns were checked under a light microscope and photographed every 2 h.

### 2.2. Bioinformatic Analyses

Online analyses were conducted to retrieve the sequences of the *MaSep1* gene and the MaSep1 protein (https://www.ncbi.nlm.nih.gov/, accessed on 15 December 2021) and predict isoelectric point (pI) and molecular weight (https://web.expasy.org/protparam/, accessed on 15 December 2021), conserved domains (https://www.ncbi.nlm.nih.gov/Structure/cdd/wrpsb.cgi, accessed on 15 December 2021), and the putative target DNA motif of MaSep1 (https://jaspar.genereg.net/, accessed on 10 December 2022). The multiple sequence alignment analysis was performed using the DNAMAN program 8. The program MEGA v7.0 (www.megasoftware.net, accessed on 20 December 2021) was used to construct the neighbor-joining tree under 1000 bootstrap replicates.

### 2.3. Gene Disruption and Complementation

About 1000 bp of 5′ and 3′ flanking fragments of *MaSep1* (MAC_03141) or *MaFkh2* (MAC_06478) genes were inserted into the pK2-PB vector, respectively. The completed vectors were introduced into CQMa102 using the *Agrobacterium*-mediated method, respectively. Potential mutants were selected using Czapek–Dox medium supplemented with 500 µg/mL glyphosate (Sigma, St. Louis, MO, USA), which were subsequently validated by PCR. The disruption strain of *MaAce2* (MAC_04654) has been constructed previously [34]. To rescue *MaSep1* in the Δ*MaSep1* strain, the native promoter and open reading frame (ORF) of *MaSep1* were incorporated into the pK2-Sur vector [35]. The resultant vector was introduced into the Δ*MaSep1* strain using the method described above. Complemented transformants (CP) of *MaSep1* were screened on Czapek–Dox medium with 20 μg/mL chlorimuron ethyl (Sigma, Bellefonte, PA, USA). Quantitative reverse transcription PCR (qRT-PCR) was adopted to further verify the *MaSep1* disruption and complementation strains. The above-mentioned primers are recorded in Table S1.

### 2.4. Conidial Germination and Conidiation Capacity Assays

Conidial germination was evaluated following previously established methods on 1/4 SDAY medium [36]. One hundred microliters of the suspension (1 × 10^7^ conidia/mL) were evenly spread onto 1/4 SDAY medium and incubated at 28 °C. The conidial germination rates of the WT, Δ*MaSep1*, and CP strains were examined every 2 h by taking samples using a previously described method [37]. For conidiation capacity assays, two microliters of conidial suspensions (1 × 10^6^ conidia/mL) from the WT, Δ*MaSep1*, and CP strains were placed onto 1/4 SDAY medium in a 12-well plate and grown at 28 °C. Fungal samples incubated for 3, 6, 9, 12, and 15 days were harvested in each well with 0.05% Tween 80 to disperse the conidia evenly by vortexing. Conidia were examined using a hemocytometer.

### 2.5. Stress Tolerance Assays

Fungal resistance to ultraviolet (UV-B) irradiation and heat shock were evaluated as previously described [38]. In short, 100 μL of the suspensions (1 × 10^7^ conidia/mL) were spread onto 1/4 SDAY medium and treated by UV-B irradiation for 0.5, 1.0, 1.5, 2.0, and 2.5 h, or heat shock at 42.5 °C for 2.0, 4.0, 6.0, 8.0, and 10.0 h, followed by incubation at 28 °C for 20 h. The germination of conidia at each time point after different treatments was recorded and statistically analyzed. Three replicates for each time point were performed [35]. For spot assays, mature conidia from WT, Δ*MaSep1*, and CP strains were prepared at a concentration of 1 × 10^6^ conidia/mL. Then, 2 μL of conidial suspensions were inoculated onto 1/4 SDAY medium added with sorbitol (SOR), NaCl, H_2_O_2_, SDS, and Congo red (CR), respectively. The inoculated plates were allowed to dry and then incubated upside down at 28 °C. After a 6-day incubation, the colony sizes were measured.

### 2.6. DNA Damage and Repair

One hundred microliters of the suspension (5 × 10^7^ conidia/mL) from each fungal strain was spread onto 1/4 SDAY medium and subjected to 1350 mW/m^2^ UV light for 1.5 h or to a 42.5 °C water bath for 8 h. DAPI was employed to stain the DNA of each strain and the conidia were photographed under laser confocal scanning microscope (LSCM) to observe the nucleic acid damage. The remaining UV/heat shock-treated conidia were incubated at 28 °C for 20 h, and then taken out for DAPI staining and photographing as stated above.

### 2.7. Pathogenicity Assays

Locusts, *Locusta migratoria manilensis*, were reared at 28 °C with a 16:8 h (light–dark) cycle in our lab. The bioassays with 5th instar nymphs were conducted by a previous method [39]. Conidial suspensions (1 × 10^7^ conidia/mL) were prepared with paraffin oil using mature conidia from WT, Δ*MaSep1*, and CP strains, and 5 μL of conidial suspensions were dripped onto the locusts with paraffin oil as the blank control. Thirty locusts were dripped in each group. Locusts were raised on fresh corn leaves and counted every 12 h.

### 2.8. qRT-PCR and RNA-Seq

Total RNA was obtained from fungal cultures on SYA medium using the Ultrapure RNA Kit (CWBIO, Beijing, China). RNA quality and quantity were examined using a Nano Drop spectrophotometry and an Agilent 2100 Bioanalyzer (Thermo Fisher Scientific, Waltham, MA, USA). qRT-PCR was conducted using SYBRPrime qPCR Set (BAOGUANG, Chongqing, China). The transcriptional levels of target genes were calculated using the 2^−ΔΔCt^ method [40]. The *gpdh* gene (MAC_09584) was employed as the internal control. For the transcriptome analysis, samples of Δ*MaSep1* and WT grown on SYA medium for 8, 10, and 12 h were harvested to extract RNA for RNA-Seq with three biological replicates, respectively. RNA library construction and RNA sequencing were conducted on a BGIseq500 platform by BGI (Wuhan, China). Clean reads were obtained using SOAPnuke software (v1.5.2) [41] and mapped to the reference genome of *M. acridum* (SUB13250283) using HISAT2 software (v2.0.4) [42]. Gene expression levels were calculated using RSEM software (v1.2.12) [43]. Differentially expressed genes (DEGs) were defined by the threshold of |Log2 ratio| ≥ 1 and Qvalue ≤ 0.05. DEGs were annotated through Gene Ontology (GO) enrichment analysis (http://www.geneontology.org/, accessed on 16 March 2022) and Kyoto Encyclopedia of Genes and Genomes (KEGG) enrichment analysis (https://www.genome.jp/kegg, accessed on 16 March 2022).

### 2.9. Data Analyses

The primers were designed using Primer Premier 5 software. The specific primers used in qRT-PCR were designed using Beacon Designer 2.0 software (Bio-Rad, Hercules, CA, USA) (Table S1). The Shapiro–Wilk test and Levene’s test were applied to check the normality and homogeneity of variances. Statistical analysis of the experimental data was conducted using one-way analysis of variance (ANOVA) in SPSS 22.0. Graphpad Prism 5 and Adobe Photoshop 2022 software were used for image processing.

## 3. Results

### 3.1. Features of MaSep1

**Figure 1:**
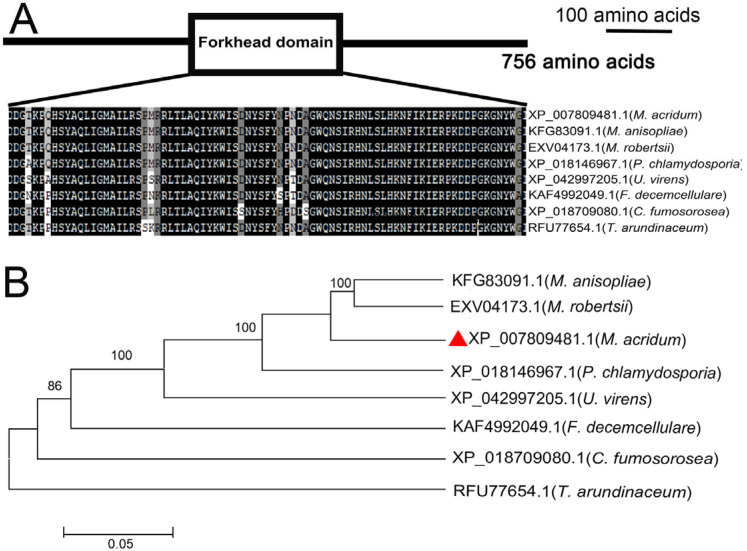
Features of MaSep1. (**A**) The location and multiple sequence alignment analysis of Forkhead domain. (**B**) Phylogenetic tree of Sep1 in different filamentous fungi. The phylogenetic tree was performed using MEGA software. *M. acridum* (XP_007809481.1), *Ustilaginoidea virens* (XP_042997205.1), *M. anisopliae* (KFG83091.1), *M. robertsii* (EXV04173.1), *Pochonia chlamydosporia* (XP_018146967.1), *Fusarium decemcellulare* (KAF4992049.1), *Cordyceps fumosorosea* (XP_018709080.1), and *Trichoderma arundinaceum* (RFU77654.1). Red triangle represented the Sep1 homologous protein in *M. acridum*.

*MaSep1*, the *Sep1* homologous gene in *M. acridum*, was obtained from the CQMa102 genome (SUB13250283). MaSep1 comprises a 2271 bp ORF, encoding a predicted 756 amino acids (82 kDa) with a pI of 9.61. Domain predictions using SMART revealed the presence of a forkhead domain in MaSep1, and the sequences of the forkhead domain displayed highly conserved in fungi (Figure 1A). Phylogenetic analysis of MaSep1 and its homologs in other fungi indicated that MaSep1 is closely related to *M. robertsii* and *M. anisopliae* (Figure 1B).

### 3.2. Deletion of MaSep1 Affected Conidial Germination and Conidial Production, but Not Virulence

**Figure 2:**
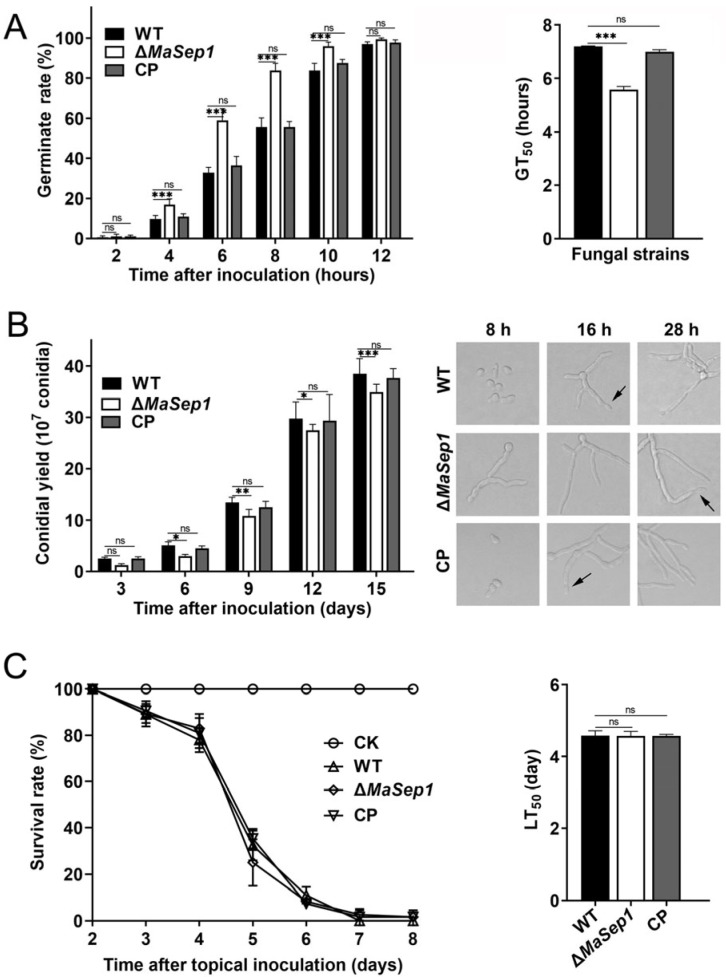
Conidial germination and conidial production. (**A**) Conidial germinations. (**B**) Conidiation. Black arrows represent conidia on the conidiophores. (**C**) The survival rates of the locusts, *L. migratoria manilensis*, and LT_50_ of the fungal strains were evaluated following topical inoculation with 5 μL conidial suspensions. Error bars indicate the standard error of the mean (SEM) from triplicated assays. ns, not significant, *p* > 0.05. *, *p* < 0.05; **, *p* < 0.01; ***, *p* < 0.001.

To reveal the roles of *MaSep1* in conidial germination and conidial production in *M. acridum*, the conidial germination rates and yields of WT, Δ*MaSep1*, and CP strains were assessed on 1/4 SDAY medium. The conidial germination rate of Δ*MaSep1* was markedly higher compared to the WT and CP strains. Approximately 60% of conidia from Δ*MaSep1* had germinated, whereas less than 40% of conidia from the WT and CP strains had germinated at 6 h after incubation (Figure 2A). The average 50% germination time (GT_50_) of Δ*MaSep1* was remarkably lower relative to the WT and CP strains (Figure 2A). On 1/4 SDAY medium, the conidial yield of Δ*MaSep1* was markedly lower compared to the WT and CP strains. (Figure 2B). Bioassays conducted by topical inoculation showed that the half-lethal time (LT_50_) of Δ*MaSep1* did not differ significantly from the WT and CP strains (Figure 2C), indicating that disruption of *MaSep1* did not affect the virulence of *M. acridum*.

### 3.3. Deletion of MaSep1 Affects Multiple Stress Tolerances in M. acridum

**Figure 3:**
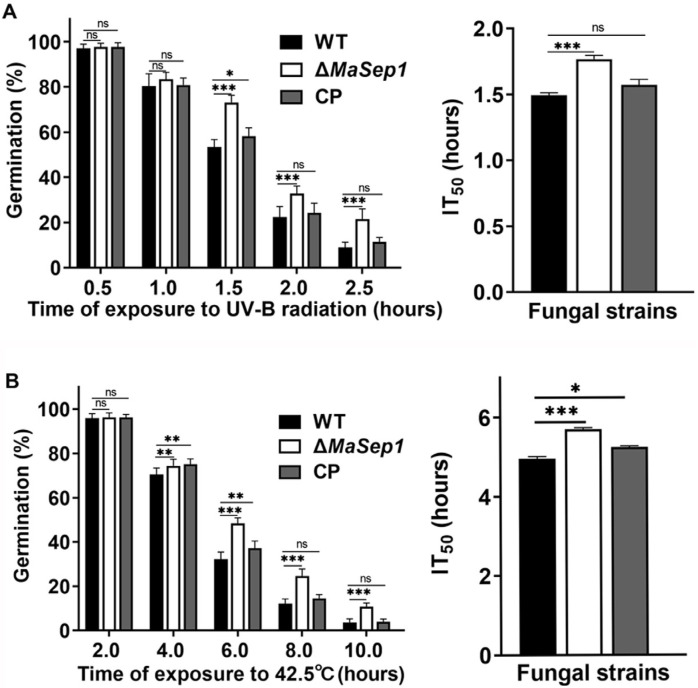
Deletion of *MaSep1* increased the fungal tolerances to UV-B and heat shock. (**A**) Conidial germination rates and IT_50_ of fungal strains after UV-B irradiation at 1350 mW/m^2^ for 0.5, 1.0, 1.5, 2.0, and 2.5 h. (**B**) Conidial germination rates and IT_50_ of fungal strains after heat shock at 42.5 °C for 2, 4, 6, 8, and 10 h. Error bars indicate the SEM from triplicated assays. ns, not significant, *p* > 0.05. *, *p* < 0.05; **, *p* < 0.01; ***, *p* < 0.001.

To reveal the function of *MaSep1* in stress tolerances, we examined the germination rates of conidia following heat shock and UV-B irradiation. The findings showed that Δ*MaSep1* demonstrated notably higher conidial germination rates after UV-B irradiation for 1.5, 2.0, and 2.5 h. Additionally, the half-inhibition time of germination (IT_50_) for the Δ*MaSep1* strain was considerably higher in comparison with those of the WT and CP (Figure 3A). Similarly, Δ*MaSep1* exhibited significantly increased conidial germination rates compared to the WT and CP strains after 4, 6, 8, and 10 h of treatments at 42.5 °C (Figure 3B).

**Figure 4:**
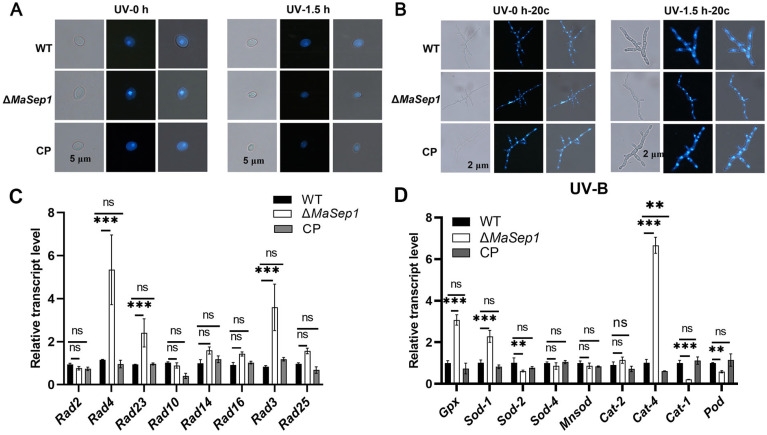
Analysis of DNA damage and repair under UV-B irradiation. (**A**) DNA damage was observed after UV-B irradiation. “UV-0 h” means no UV-B irradiation; “UV-1.5 h” means UV-B irradiation for 1.5 h. (**B**) DNA repair was observed after UV-B irradiation. “UV-0 h-20c” means grown for 20 h at 28 °C without UV-B irradiation; “UV-1.5 h-20c” means grown for 20 h at 28 °C after UV-B irradiation for 1.5 h. (**C**) Transcriptional expression of key genes in the NER pathway in different strains after UV-B irradiation. (**D**) The relative transcriptional expression of ROS scavenging genes in different strains after UV treatment. Error bars indicate the SEM from triplicated assays. ns, not significant, *p* > 0.05. **, *p* < 0.01; ***, *p* < 0.001.

To assess DNA damage in conidia from different fungal strains after UV-B irradiation, conidia treated with 1.5 h of UV-B irradiation and conidia incubated on 1/4 SDAY medium for 20 h after 1.5 h of UV-B irradiation were stained with DAPI, respectively. The results indicated that the DNA in fungal strains was severely damaged to a comparable extent and exhibited a diffuse pattern after 1.5 h of treatment with UV-B irradiation (Figure 4A). However, the conidia of Δ*MaSep1* germinated to form long mycelium, and the damaged DNA was repaired and became aggregated again after 20 h of incubation, while the conidia of WT and CP still had the ability to germinate but their DNA was still in a diffuse state (Figure 4B). qRT-PCR showed that the expression of most of the genes (*Rad3*, *Rad4*, *Rad23*) in the NER pathway was significantly upregulated in Δ*MaSep1* (Figure 4C). To understand the differences in the ROS scavenging ability of the WT, Δ*MaSep1,* and CP strains, conidia incubated for 20 h after UV-B irradiation were collected to detect the transcriptional levels of some genes related to ROS scavenging by qRT-PCR. The findings displayed that most of the oxidoreductase-encoding genes (*Gpx*, *Sod*, and *Cat*) involved in ROS scavenging were significantly upregulated in Δ*MaSep1* (Figure 4D).

**Figure 5:**
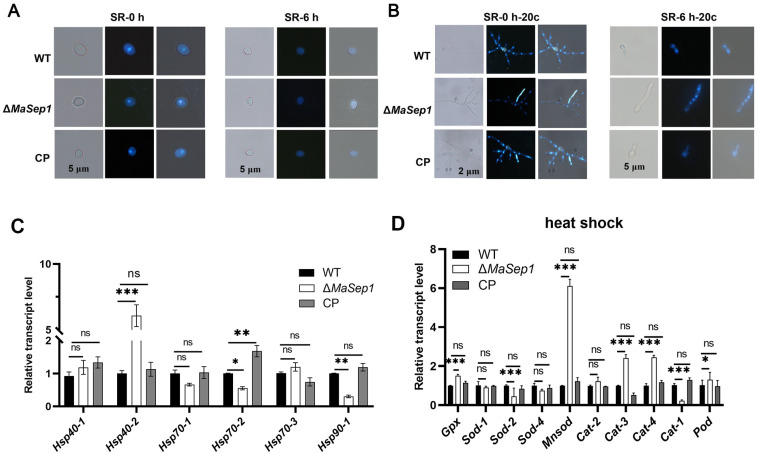
Analysis of DNA damage and repair under heat shock. (**A**) DNA damage was observed after thermo treatment. “SR-0 h” means no heat shock; “SR-6 h” means heat shock for 6 h. (**B**) DNA repair was observed after heat shock. “SR-0 h-20c” means grown for 20 h at 28 °C without heat shock; “SR-6 h-20c” means grown for 20 h at 28 °C after heat shock for 6 h. (**C**) The relative transcriptional expression levels of heat shock protein-encoding genes among different strains after heat shock. (**D**) The relative transcriptional expression of ROS scavenging genes in different strains after heat shock. Error bars indicate the SEM from triplicated assays. ns, not significant, *p* > 0.05. *, *p* < 0.05; **, *p* < 0.01; ***, *p* < 0.001.

To explore the effect of heat shock stress on the DNA of WT, Δ*MaSep1,* and CP strains, conidia exposed to heat shock for 6 h and conidia incubated for 20 h after 6 h of heat shock were, respectively, collected and stained using DAPI to observe the DNA damage. The results showed that after 6 h of exposure to heat shock, DNA in all fungal strains was severely damaged in a diffuse manner (Figure 5A). However, after 20 h of incubation, the conidia of Δ*MaSep1* germinated to form long mycelium and the damaged DNA was repaired and became aggregated again, while the conidia of WT and CP still had the ability to germinate but their DNA was still in a diffuse state (Figure 5B). qRT-PCR showed that *Hsp40-2* in Δ*MaSep1* was significantly upregulated (Figure 5C). Similar to the UV-B treatments, conidia from the WT, Δ*MaSep1,* and CP strains incubated for 20 h after heat shock were collected to detect the transcriptional levels of some genes related to ROS scavenging by qRT-PCR. The results displayed that most of the oxidoreductase-encoding genes (*Gpx*, *Sod*, *Cat*) involved in ROS scavenging were significantly upregulated in Δ*MaSep1* (Figure 5D).

**Figure 6:**
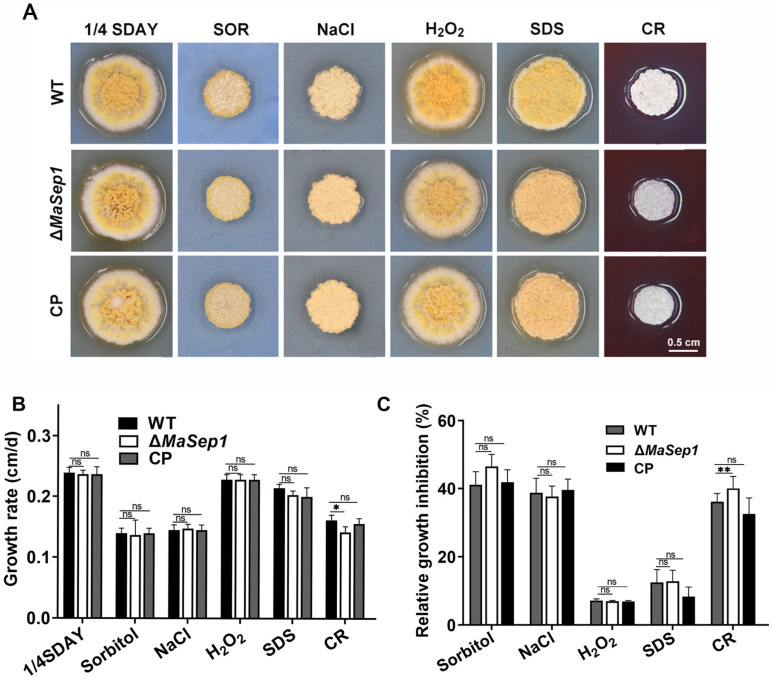
Stress tolerance assays. (**A**) Colonies of different fungal strains on 1/4 SDAY medium with SOR, NaCl, H_2_O_2_, SDS, and CR for 6 days. (**B**) Growth rates of different fungal strains. (**C**) Relative growth inhibition rates of different fungal strains. Error bars indicate the SEM from triplicated assays. ns, not significant, *p* > 0.05. *, *p* < 0.05; **, *p* < 0.01.

Additionally, fungal responses to oxidative stress (H_2_O_2_), cell wall stress (CR), cell membrane stress (SDS), and osmotic stress (SOR and NaCl) were evaluated. The findings demonstrated that the colony morphology of Δ*MaSep1* exhibited insignificant differences compared to the WT and CP strains, except when grown on 1/4 SDAY medium added with CR (Figure 6A). The disruption of *MaSep1* significantly reduced the fungal growth rate on 1/4 SDAY containing the CR (Figure 6B). For the relative inhibition rate, Δ*MaSep1* was more sensitive to CR than WT and CP (Figure 6C).

### 3.4. MaSep1 Is Required for Microcycle Conidiation of M. acridum

**Figure 7:**
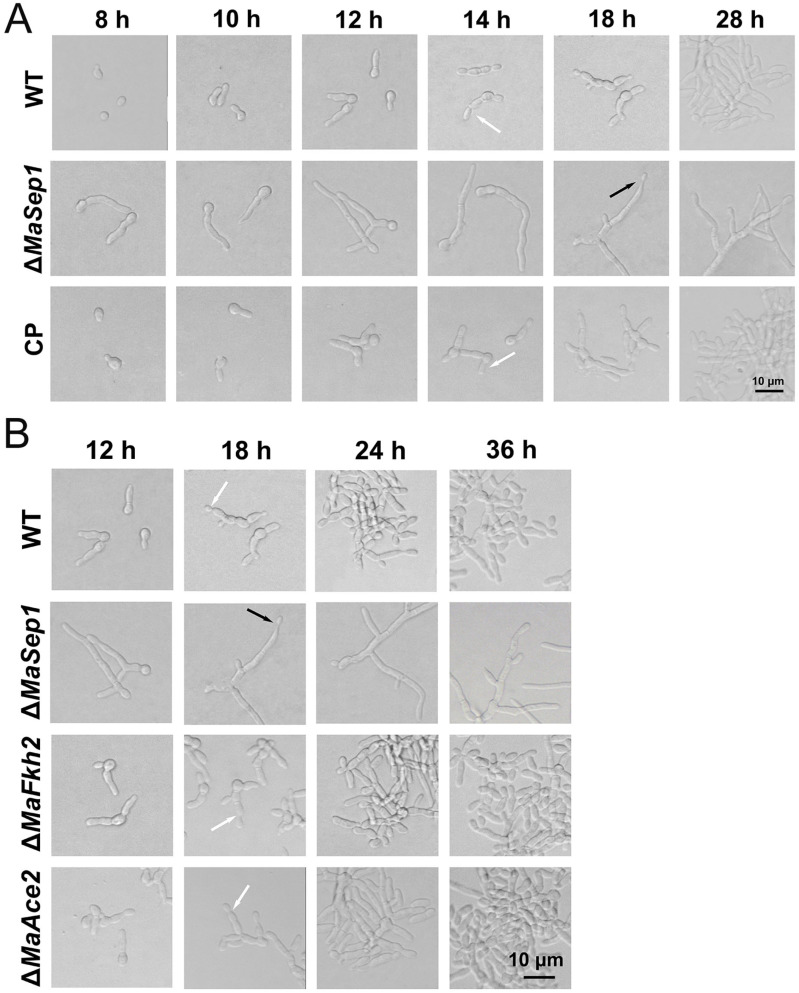
Conidiation pattern analyses of fungal strains grown on SYA medium. (**A**) The conidiation patterns of WT, Δ*MaSep1,* and CP strains. (**B**) The conidiation patterns of WT, Δ*MaSep1*, Δ*MaFkh2,* and Δ*MaAce2* strains. Black arrows represent normal conidiation. White arrows represent microcycle conidiation.

To examine the impact of MaSep1 on the conidiation pattern of *M. acridum*, the conidiation process of Δ*MaSep1* was monitored on the SYA medium. On the SYA medium, Δ*MaSep1* displayed microcycle conidiation, while WT and CP exhibited normal conidiation (Figure 7A). Sep1, Ace2, and Fkh2 jointly regulate cell division in *S. pombe* [27]. To reveal the roles of Ace2 and Fkh2 on microcycle conidiation in *M. acridum*, *MaFkh2* and *MaAce2* were deleted, respectively, in *M. acridum,* and the conidiation of the *MaFkh2*-disruption strain (Δ*MaFkh2*) and the *MaAce2*-disruption strain (Δ*MaAce2*) was observed on the SYA medium. The results showed that WT, Δ*MaFkh2,* and Δ*MaAce2* performed microcycle conidiation on SYA medium, while Δ*MaSep1* showed normal conidiation on SYA medium (Figure 7B). These results suggest that MaSep1 contributed to the microcycle conidiation independent of MaFkh2 and MaAce2 in *M. acridum*.

### 3.5. Identification of the DEGs Regulated by MaSep1 Using RNA-Seq

**Figure 8:**
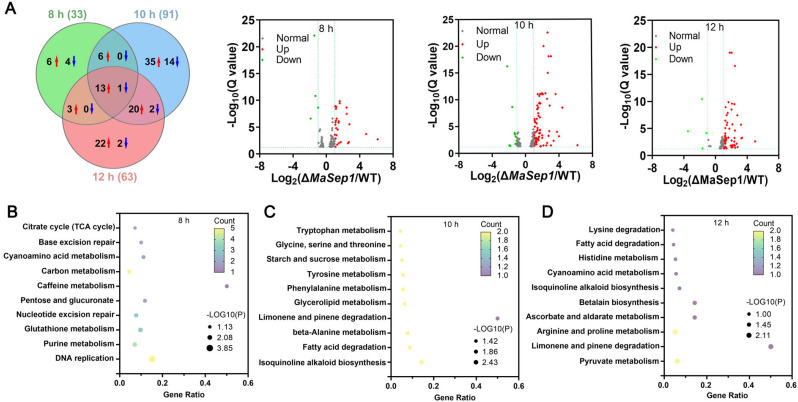
The analyses of RNA-seq data. (**A**) The number of DEGs. Red arrows represented up-regultaion, blue arrows represented down-regulation. The KEGG pathway enrichment at 8 (**B**), 10 (**C**), and 12 h (**D**).

RNA-seq was performed to recognize DEGs between the Δ*MaSep1* and WT strains cultivated on SYA medium for 8, 10, and 12 h. In total, 127 DEGs were found between the Δ*MaSep1* and WT strains (Table S2). Of these DEGs, 112 DEGs were upregulated and 15 DEGs were down-regulated in Δ*MaSep1* (Figure 8A). At 8 h, 29 DEGs showed upregulation, while 4 DEGs were down-regulated in Δ*MaSep1*. The KEGG pathways of these DEGs were significantly enriched in DNA replication, purine metabolism, glutathione metabolism, nucleotide excision repair, pentose and glucuronate interconversions, carbon metabolism, cyanoamino acid metabolism, base excision repair, and TCA cycle (Figure 8B). At 10 h, 80 DEGs were upregulated and 11 DEGs were down-regulated in Δ*MaSep1*. The KEGG pathways of these DEGs were significantly enriched in fatty acid degradation, glycerolipid metabolism, β-alanine metabolism, phenylalanine metabolism, isoquinoline alkaloid biosynthesis, starch and sucrose metabolism, limonene and pinene degradation, tyrosine metabolism, glycine, serine and threonine metabolism, and tryptophan metabolism (Figure 8C). At 12 h, 59 DEGs were upregulated and 4 DEGs were down-regulated in Δ*MaSep1*. The KEGG pathways of these DEGs were significantly enriched in limonene and pinene degradation, pyruvate metabolism, arginine and proline metabolism, ascorbate and aldarate metabolism, betalain biosynthesis, isoquinoline alkaloid biosynthesis, cyanoamino acid metabolism, histidine metabolism, fatty acid degradation, and lysine degradation (Figure 8D).

**Figure 9:**
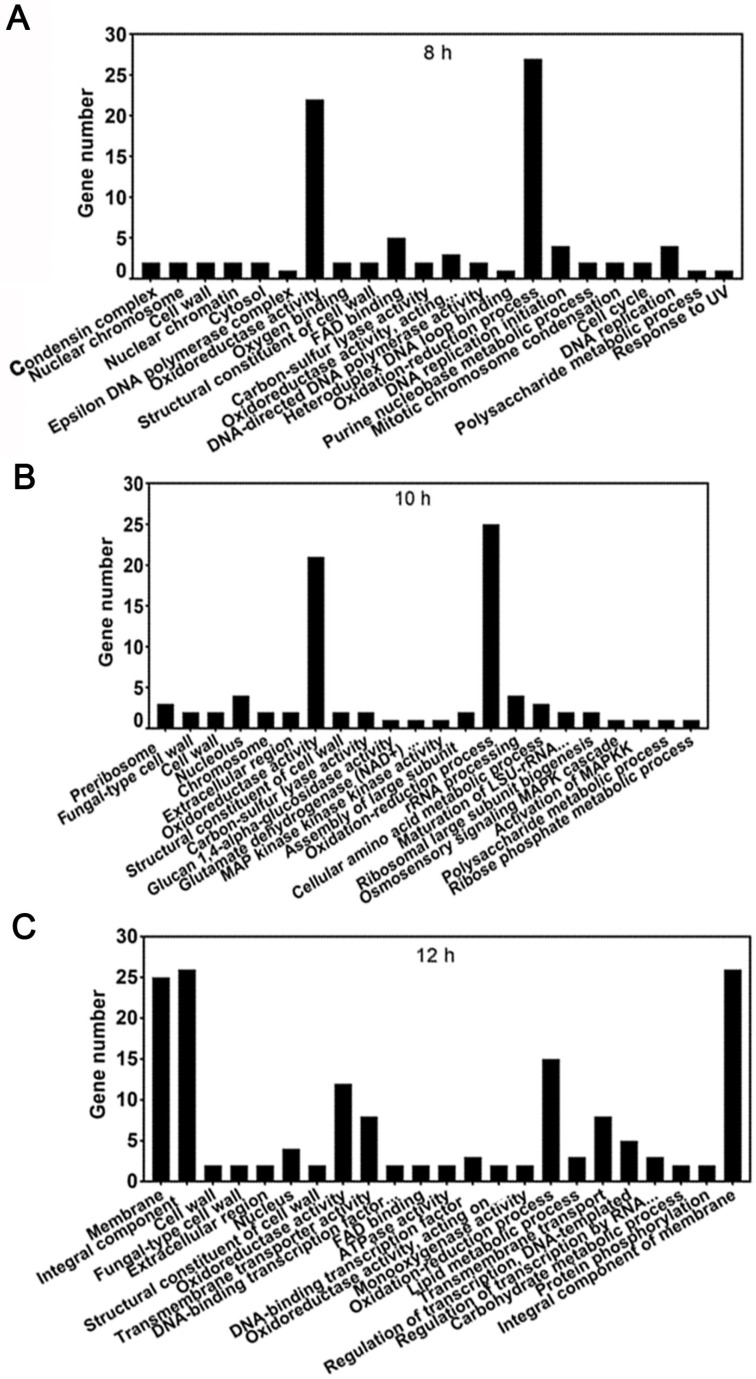
Significant enrichment of DEGs on GO terms at 8 (**A**), 10 (**B**), and 12 h (**C**).

In GO function enrichment analysis, DEGs were mainly enriched in biological processes, cellular components, and molecular functions. At 8 h, DEGs were mainly enriched in MCM complex, oxidoreductase activity, FAD binding, oxidation-reduction processes, and DNA replication (Figure 9A). At 10 h, DEGs were mainly enriched in the nucleus, preribosome, nucleolus, oxidoreductase activity, oxidation-reduction processes, rRNA processing, and cellular amino acid metabolic processes (Figure 9B). At 12 h, DEGs were mainly enriched in integral components of membrane, membrane, nucleus, oxidoreductase activity, transmembrane transporter activity, oxidation-reduction processes, transmembrane transport, regulation of transcription, and DNA-templated processes (Figure 9C).

Bioinformatics analysis showed that there were 105 putative target genes of MaSep1 in the 127 DEGs identified by RNA-seq during the conidiation pattern shift of *M. acridum* (Table S3). Of these putative target DEGs, 26 genes were related to conidiation in Δ*MaSep1* (Table S4). Fourteen of those participated in cell wall formation, comprising cell wall protein (MAC_06850 and MAC_06624), hydrophobic protein (MAC_04376 and MAC_07330), glucosidase (MAC_00623, MAC_04525, and MAC_08796) and NLPC/P60-like cell wall peptidase (MAC_06296). Nine of those participated in hyphal development and conidiation, comprising transcription factors (MAC_00186), ammonium osmotic enzyme MEPC (MAC_03001), and MFS family transporters (MAC_06625 and MAC_08197). Three of those were involved in the cell cycle, RNA binding protein MSSP-2 (MAC_01348), and reverse transcriptase protein (MAC_09371). These results indicate that MaSep1 governs the conidiation pattern shift by regulating the expression of genes associated with cell wall formation, conidiation, and cell cycle in *M. acridum*.

## 4. Discussion

In this investigation, we identified the forkhead transcription factor MaSep1 in *M. acridum* and explored its functions. The disruption of *MaSep1* led to accelerated conidial germination, reduced conidial production, and enhanced resistance to UV-B irradiation and heat shock while fungal virulence remained unaffected. More importantly, *M. acridum* could not perform microcycle conidiation when *MaSep1* was disrupted. Furthermore, some DEGs involved in microcycle conidiation were identified in Δ*MaSep1* by RNA-seq.

For insect pathogenic fungi, stress tolerance is a crucial factor for survival and infectivity in hosts [44]. In this study, Δ*MaSep1* showed significantly increased tolerances to UV-B irradiation and heat shock, and the tolerance of Δ*MaSep1* to the cell wall disruptor Congo red was decreased. The stress tolerance of fungi is closely related to cell wall integrity [45]. Congo red can bind to β-1,3-glucan specifically, thus hindering the normal formation of cell walls [46]. NER is the pathway to remove bulky lesions from UV-B irradiation, and NER plays a crucial role in fungal tolerance to UV-B irradiation [47]. The *Rad25* gene of *S. cerevisiae* is essential for NER after UV-B irradiation [48]. RAD4 exhibits very high activity in NER after UV irradiation in *M. robertsii* [49]. Thus, the enhanced ability of DNA repair increased the tolerance of *M. acridum* to UV-B irradiation. HSPs are a class of proteins induced by high temperatures in fungi, which can help proteins fold correctly and restore the original spatial conformation and biological activity, which plays a crucial role in fungal heat tolerance [50]. Hsp70 is widely present in fungi, and its expression is influenced by temperature [51]. The high expression of genes encoded HSPs after heat shock treatment may be responsible for the heat shock tolerance of fungi [52].

Large amounts of reactive oxygen species (ROS) were induced after UV-B irradiation and heat shock treatments. The ability to clean ROS reflects the tolerance to abiotic stress [53]. ROS are usually produced as by-products of metabolic processes in living organisms [54]. Although ROS play an important role in various cellular processes, excessive ROS accumulation can cause irreversible damage to cellular biomacromolecules [55]. Therefore, ROS scavenging capacity is a major determinant of fungal stress response. The enzyme system consisting of POD, CAT, SOD, and GPX is an important weapon for ROS scavenging [47]. In this study, the upregulated expression of genes related to DNA excision repair, HSPs, and ROS scavenging in Δ*MaSep1* may be related to the enhanced tolerances of Δ*MaSep1* to UV irradiation and heat shock.

Sep1 involved in cell separation was first reported in *S. pombe*. The disruption of *Sep1* hindered the separation of the cell septum and resulted in impaired cell separation in *S. pombe* [25,56]. In this study, MaSep1, the homolog of Sep1 in *S. pombe*, was found to be required for microcycle conidiation in *M. acridum*. The expression of *Ace2* is regulated by a transcriptional complex composed of Sep1 and Fkh2 [27]. Sep1p and Ace2p transcriptionally regulate the initiation of the division of daughter cells following cytoplasmic division [29]. Sep1 is required for Fkh2 activity, and stimulation of forkhead transcription factors, Fkh2p and Sep1p, triggers the mitotic gene expression in *S. pombe* [57]. In this research, we discovered that Δ*MaSep1* displayed normal conidiation on SYA medium, but the Δ*MaAce2* and Δ*MaFkh2* strains performed microcycle conidiation, which is similar to WT, demonstrating that MaSep1 contributed to microcycle conidiation in *M. acridum* independent of MaAce2 and MaFkh2. RNA-seq data show that MaSep1 regulates conidiation pattern shift mainly by governing the expression of genes associated with cell wall synthesis, the cell cycle, and cell separation. Among the DEGs we studied, some are involved in cell development. A gene for hydrophobin (MAC_04376) was upregulated in Δ*MaSep1*. In *M. brunneum*, the growth of the deletion strain is slowed and conidiation is delayed [58]. The MFS transporter gene (MAC_06625), critical for fungal hyphal morphology, exhibited upregulation [59]. Moreover, some DEGs relevant to cell wall synthesis were identified. A gene for cell wall protein (MAC_06850) was upregulated in Δ*MaSep1* and was critical for the integrity of fungal cell walls [60]. A GNAT family acetyltransferase (MAC_04676), which is involved in chitin metabolism and cell wall rearrangement in fungi, was upregulated [61]. In addition, several genes related to the cell cycle and cell division showed differential expression. Upregulated genes included those encoding integral membrane proteins (MAC_07739, MAC_06568, and MAC_01217), which play crucial roles in linking the plasma membrane to the actomyosin ring and the assembly of the segmentation septum machinery in fission yeast [62].

In conclusion, MaSep1 negatively regulates the UV- and thermo-tolerances of *M. acridum* by influencing the expression of some genes associated with DNA damage repair and heat shock response, and mediates the conidiation pattern shift of *M. acridum* by governing some genes associated with conidiation, cell division and cell wall formation. These results will provide theoretical insights for further elucidating the molecular mechanism of stress tolerances and microcycle conidiation in filamentous fungi.

## Data Availability

RNA-seq data have been deposited in the NCBI BioProject database (accession number: PRJNA974819).

## Supplementary Materials

The following supporting information can be downloaded at: https://www.mdpi.com/article/10.3390/jof10080544/s1, Figure S1: The disruption and complementation of *MaSep1*. (A) The knockout and complement schematic diagram of *MaSep1*. (B) Transcription level analysis of *MaSep1* in WT, Δ*MaSep1,* and CP strains. *MaSep1* expression in WT as control. Figure S2: PCR was used to verify the disruption of *MaFkh2*. (A) Fkh2-VF/Pt-R was used to verify the left arm of Δ*MaFkh2*. (B) Fkh2-VR/Bar-F was used to verify the right arm of Δ*MaFkh2*. M: DNA marker. Lanes 25–32: different mutants. Δ*MaFkh2*-27# was used for observation of conidiation pattern. Table S1: Primers used in this work. Table S2: Description of these DEGs. Table S3: 105 putative target genes of MaSep1. Table S4: DEGs involved in conidiation pattern shift. References [58,59,60,62,63,64,65,66,67,68,69,70,71,72,73,74,75,76,77,78,79,80,81] are cited in the Supplementary Materials.
