# Supplementary material for: The Forkhead Box Gene, MaSep1, Negatively Regulates UV- and Thermo-Tolerances and Is Required for Microcycle Conidiation in Metarhizium acridum
Source: J Fungi (Basel). 2024 Aug 2;10(8):544. doi: 10.3390/jof10080544 (PMC11355920; doi:10.3390/jof10080544)
Supplement: Supplementary file 1 [file jof-10-00544-s001.zip › jof-3125144-supplementary.pdf]

The forkhead box gene, *MaSep1*, negatively regulates stress tolerances to UV irradiation and heat shock and is required for microcycle conidiation in *Metarhizium acridum*

Tiantian Song[1,2,3#], Chan Li[1,2,3#], Kai Jin[1,2,3]*, Yuxian Xia[1,2,3]*

1 Genetic Engineering Research Center, School of Life Sciences, Chongqing University, Chongqing 401331, PR China; 202226021001@cqu.edu.cn; 202026021001@cqu.edu.cn.

2 Chongqing Engineering Research Center for Fungal Insecticide, Chongqing 401331, PR China

3 Key Laboratory of Gene Function and Regulation Technologies Under Chongqing Municipal Education Commission, Chongqing 401331, PR China

# These authors contributed equally to this work.

* Correspondence: jinkai@cqu.edu.cn and yuxianxia@cqu.edu.cn

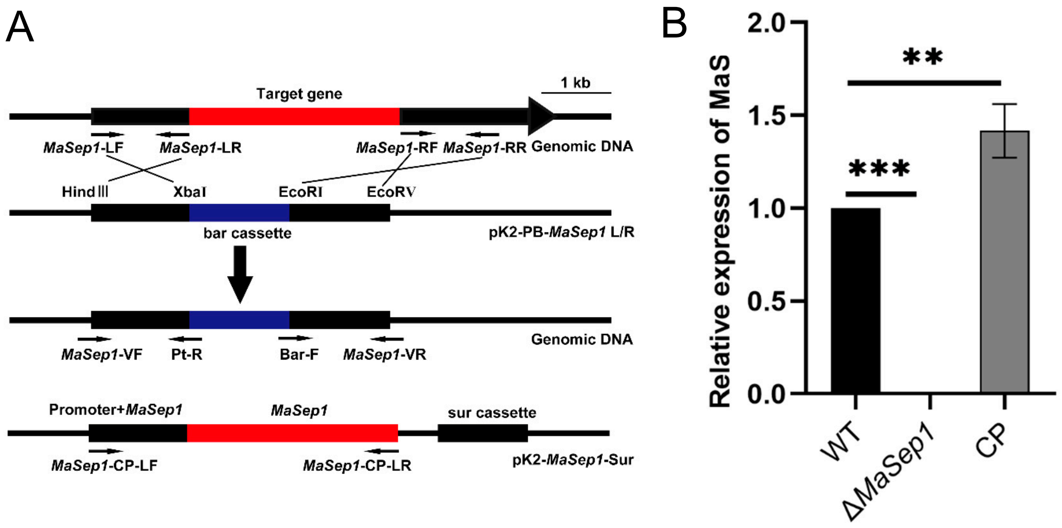

Figure S1. The disruption and complementation of *MaSep1*. (A)The knockout and complement schematic diagram of *MaSep1*. (B) Transcription level analysis of *MaSep1* in WT, Δ*MaSep1* and CP strains. *MaSep1* expression in WT as control.

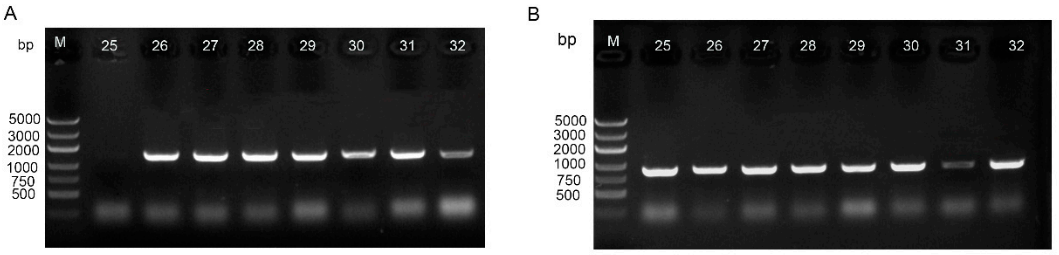

Figure S2. PCR was used to verify the disruption of *MaFkh2*. (A) Fkh2-VF/Pt-R was used to verify the left arm of Δ*MaFkh2*. (B) Fkh2-VR/Bar-F was used to verify the right arm of Δ*MaFkh2*. M: DNA marker. Lanes 25-32: different mutants. Δ*MaFkh2*-27# were used for observation of conidiation pattern.

Table S1. Primers used in this work.

Primers	Sequences (5′- 3′)	Remarks
MaSep1-LF	CTGGCGAAAGTGGTGAATCT	Used to clone the 5′ end of MaSep1
MaSep1-LR	GGTATGACTGCCGAGTGATGT	
MaSep1-RF	GATTTGCCACGGTCTGTTCT	Used to clone the 3′ end of MaSep1
MaSep1-RR	CTCCCGCATGAGGATCTACT	
Pt-R	CAGCCAAGCCCAAAAAGTG	
Bar-F	GCTCTACACCCACCTGCT	Used for screening the MaSep1-disruption transformants
MaSep1-VF	TCTGGAACGTGGAACCCG	
MaSep1-VR	CTTTGCCAACGCCCATAA	
MaSep1-CPF	TGGGCAATGGTCTCCTCTC	Used to form pK2- MaSep1-EGFP-SUR vector
MaSep1-CPR	GAAGGCGGTAGAATAGCTCC	
CP-VF	CGATCCCAATCTCAGACAAC	Used for screening the MaSep1-complement transformants
EGFP-VR	CGATGCGGTTCACCAGG	
MaSep1-qF	AATGGACGACAGTGGTTA	Used to analyze the expression of MaSep1 by qRT-PCR (E=114%)
MaSep1-qR	CGATCAGCCTCAGAAGTA	
MaFkh2-LF	TTTGCCGAGTTGCTGATG	Used to clone the 5’ end of MaFkh2
MaFkh2-LR	CAAGCGTAGCGTGGATGT	
MaFkh2-RF	AGGTGCGGTTGGTTCGTC	Used to clone the 3’ end of MaFkh2
MaFkh2-RR	CTGCTTCAATGCGGAGGG	
MaFkh2-VF	TGGGATTGAATGGGTGTACG	Used for screening the MaSep1-disruption transformants
MaFkh2-RR	GGTGACAAACCCGAGCA	
Rad2-QF	AGGAAGTGGTAGTGGCAACG	Used to verify the transcriptional expression of genes in NER pathway(E=103%)
Rad2-QR	GAGCTGCTGAAACTGAGGGT	
Rad3-QF	ATCACTCCTGACGGCCTAGT	(E=114%)
Rad3-QR	CTTCTGAGACTTTGCCCCGT	
Rad4-QF	AAATGCGAAGGAAGCAGGGA	(E=102%)
Rad4-QR	TCGAACGAGTTGCTGCAGAT	
Rad10-QF	CAGCCCGTACCCCAAAGAAT	(E=106%)
Rad10-QR	GCAGGTTGTGAGTCCCATGA	
Rad14-QF	ACATGATGCTGTTCCTGCGA	(E=116%)
Rad14-QR	TCTGTTCGAGTCTTGCGCTT	
Rad16-QF	AAACACGACTTTTGCCGAGC	(E=109%)
Rad16-QR	ATGCGTTGTCTGAACGGAGT	
Rad23-QF	AGGAAGTGGTAGTGGCAACG	(E=105%)
Rad23-QR	GAGCTGCTGAAACTGAGGGT	
Rad25-QF	CAAGAGGTCACGCACTCCTT	(E=106%)
Rad25-QR	CCACTCTTGGCTCGACCATT	
Hsp40-1-QF	GGCGAAGCGGATCAATTTCC	Used to verify the transcriptional expression of genes (E=107%)
Hsp40-1-QR	CATACCTTCGCCAGCGATCT	

qHSP40-2-F	CACGACCGAGTTTCAGTGGA	(E=106%)
qHSP40-2-R	TGAGAGTGACAGTCGTTGGC	
qHSP70-1-F	TGTCGATTTGGGCACCACTT	(E=105%)
qHSP70-1-R	GTAGACAATGGCGGTTGGGA	
qHSP70-2-F	GCTTGATGTTGCCCCTCTCT	(E=104%)
qHSP70-2-R	TGACGCTCACCCTCAAAGAC	
qHSP70-3-F	AGTCCTACATTGGCCGTGTG	(E=103%)
qHSP70-3-R	CTTAAGGGCAAGCTCCTGCT	
qHSP90-F	AAGAAGGTTGAGGCTGACGG	(E=103%)
qHSP90-R	GAGCTTGTGAATGCGCTCAG	
Cat1-QF	GATACGGTAACAGCAATGG	Used to verify the transcriptional levels of genes related to ROS scavenging (E=103%)
Cat1-QR	TCGGTCAAGTCAGATACG	
Cat2-QF	TTCTCGCCATCCAATCTC	(E=92%)
Cat2-QR	TCGTCGTTATTCCGTCTC	
Cat3-QF	CCTCTACTCTTCATACCTTGT	(E=110%)
Cat3-QR	GTCGCCATTCTCATTCAC	
Cat4-QF	AAGTTCCCGTTGTTCATC	(E=112%)
Cat4-QR	TTCCTGGTGAGTTGAGAG	
Pod-QF	TGTCGTGTCCAACCTGTACG	(E=108%)
Pod-QR	CGCTGTTGGCGATAATGACG	
Sod1-QF	CATGCCTATTACCTACAGTA	(E=102%)
Sod1-QR	CTTCCAGTTGATGACCTT	
Sod2-QF	AGACAAGGACACCAAGAC	(E=108%)
Sod2-QR	GGAGGCGTAGTTGAAGAT	
Sod4-QF	GGTCAAAGCTGTTGCCGTTC	(E=103%)
Sod4-QR	CGCCAAAGGTGTGAATGTGG	
MnSod-QF	CTCCTCTTCTGAACTTCC	(E=111%)
MnSod-QR	CCGAAATCCTCATTGATG	
Gpx-QF	TCGCTGACTACAAGGGCAAG	(E=105%)
Gpx-QR	CTGCATGATGGGGAAGCTGA	

Table S2. Description of these DEGs

Gene ID	log2 (8 h)	log2 (10 h)	log2 (12 h)	Up and Down regulation	GeneBank Description
MAC_00171	1.354849	1.747488	1.254830	Up	acetylcholinesterase precursor
MAC_00187	2.590361	3.037864	5.004447	Up	Maleylacetate reductase, putative
MAC_01539	1.076171	1.318690	1.259298	Up	monooxygenase, putative
MAC_02330	1.722911	1.504425	1.805380	Up	hypothetical protein
MAC_03141	-1.893041	-2.144658	-1.710290	Down	forkhead box protein L2
MAC_04376	4.762802	3.654373	2.797149	Up	hydrophobin
MAC_05784	1.135701	1.550181	1.043445	Up	lipase/esterase family protein, putative
MAC_06623	1.586745	2.585150	2.364665	Up	cytochrome P450 phenylacetate 2-hydroxylase, putative
MAC_07011	1.144803	1.678641	1.240947	Up	dienelactone hydrolase family protein
MAC_07499	2.413438	2.860290	2.764313	Up	hypothetical protein
MAC_08195	1.599824	2.234038	2.051539	Up	zinc-binding oxidoreductase ToxD, putative
MAC_08514	1.173049	1.118032	1.409213	Up	flotillin domain containing protein
MAC_08656	1.448510	2.425115	2.054106	Up	putative glyoxal oxidase precursor
MAC_00180	6.156817	6.210993	0.000000	Up	FAD dependent oxidoreductase family protein
MAC_04005	1.227009	1.320558	0.000000	Up	hypothetical protein
MAC_04635	1.491851	1.189354	0.000000	Up	glutathione-dependent formaldehyde-activating GFA
MAC_05216	1.123938	1.663678	0.000000	Up	hypothetical protein
MAC_06624	1.123938	1.663678	0.000000	Up	DUF636 domain protein
MAC_05447	1.045916	0.000000	1.242190	Up	hypothetical protein
MAC_06937	1.483241	0.000000	2.084351	Up	hypothetical protein
MAC_09524	2.440888	0.000000	2.464496	Up	glutathione S-transferase
MAC_09703	2.753015	0.000000	2.191168	Up	FAD binding domain-containing protein
MAC_00186	0.000000	4.018073	2.672235	Up	C6 transcription factor, putative
MAC_00584	0.000000	1.624207	1.322741	Up	hypothetical protein
MAC_00953	0.000000	1.456978	1.548152	Up	adhesin protein Mad2
MAC_01348	0.000000	1.531687	1.241309	Up	RNA binding protein MSSP-2
MAC_04426	0.000000	1.427538	1.217620	Up	hypothetical protein
MAC_04466	0.000000	2.149804	2.032492	Up	phosphoglycerate mutase family protein, putative
MAC_05897	0.000000	3.785451	3.232837	Up	hypothetical protein

MAC_06625	0.000000	2.000606	1.649035	Up	MFS transporter (Mch2), putative
MAC_06626	0.000000	1.777548	2.138858	Up	hypothetical protein
MAC_07182	0.000000	2.129551	1.360725	Up	hypothetical protein
MAC_07330	0.000000	3.364879	2.168779	Up	Hydrophobin-like rotein ssgA
MAC_07839	0.000000	1.741250	1.222242	Up	hypothetical protein
MAC_08196	0.000000	2.840194	2.490713	Up	hypothetical protein
MAC_08197	0.000000	2.627236	2.391814	Up	MFS monocarboxylate transporter, putative
MAC_08198	0.000000	2.722816	3.352276	Up	pyoverdine/dityrosine biosynthesis protein, putative
MAC_08199	0.000000	2.753592	2.452552	Up	aldehyde dehydrogenase
MAC_08200	0.000000	2.729319	2.409516	Up	tryptophanyl-tRNA synthetase
MAC_08201	0.000000	3.119665	3.269302	Up	hypothetical protein
MAC_08795	0.000000	1.050519	2.024778	Up	hypothetical protein
MAC_09015	0.000000	1.631960	1.308286	Up	integral membrane protein, putative
MAC_09268	0.000000	-1.296967	-1.155283	Down	delta 8-(E)-sphingolipid desaturase, putative
MAC_09686	0.000000	2.006243	1.626963	Up	hscarg dehydrogenase, putative
MAC_00623	1.179172	0.000000	0.000000	Up	beta-glucosidase
MAC_01183	-1.311523	0.000000	0.000000	Down	dihydroxyacetone kinase
MAC_01993	1.097883	0.000000	0.000000	Up	Superoxide dismutase
MAC_03702	-1.454305	0.000000	0.000000	Down	hypothetical protein
MAC_03703	-1.001611	0.000000	0.000000	Down	cryptochrome-2
MAC_05729	1.080103	0.000000	0.000000	Up	hypothetical protein
MAC_05837	2.679246	0.000000	0.000000	Up	GA4 desaturase
MAC_07788	1.149852	0.000000	0.000000	Up	hypothetical protein
MAC_08031	1.287996	0.000000	0.000000	Up	hypothetical protein
MAC_08505	1.206278	0.000000	0.000000	Up	hypothetical protein
MAC_00652	0.000000	-1.169188	0.000000	Down	hypothetical protein
MAC_00672	0.000000	1.277059	0.000000	Up	hypothetical protein
MAC_01449	0.000000	-1.072446	0.000000	Down	hypothetical protein
MAC_02060	0.000000	1.667380	0.000000	Up	hypothetical protein
MAC_02061	0.000000	1.785658	0.000000	Up	GABA permease, putative
MAC_02166	0.000000	1.451026	0.000000	Up	hypothetical protein
MAC_02311	0.000000	-1.562748	0.000000	Down	STF2-like protein
MAC_02602	0.000000	2.484354	0.000000	Up	hypothetical protein
MAC_02867	0.000000	1.793921	0.000000	Up	phosphotransferase enzyme family protein
MAC_03001	0.000000	1.810703	0.000000	Up	ammonium permease MepC
MAC_03043	0.000000	1.300188	0.000000	Up	hypothetical protein

MAC_03050	0.000000	1.070603	0.000000	Up	CFEM domain protein, putative
MAC_03761	0.000000	1.573182	0.000000	Up	cystathionine beta-lyase
MAC_03899	0.000000	1.020697	0.000000	Up	small nucleolar ribonucleoprotein complex subunit Utp15, putative
MAC_04216	0.000000	-1.204446	0.000000	Down	YT521-B-like splicing factor, putative
MAC_04372	0.000000	1.272469	0.000000	Up	ADP-ribose pyrophosphatase
MAC_04525	0.000000	1.742724	0.000000	Up	alpha-glucosidase
MAC_04532	0.000000	-1.817648	0.000000	Down	Quinate permease
MAC_04599	0.000000	1.414678	0.000000	Up	Alternative oxidase
MAC_04678	0.000000	1.086755	0.000000	Up	CipC1 protein, concanamycin induced protein C
MAC_04767	0.000000	1.725920	0.000000	Up	L-PSP endoribonuclease family protein Brt1
MAC_04883	0.000000	1.002398	0.000000	Up	hypothetical protein
MAC_04947	0.000000	1.217463	0.000000	Up	hypothetical protein
MAC_05098	0.000000	1.098380	0.000000	Up	phosphotransferase enzyme family protein
MAC_05253	0.000000	1.133223	0.000000	Up	hypothetical protein
MAC_05446	0.000000	1.165716	0.000000	Up	hypothetical protein
MAC_05716	0.000000	1.847632	0.000000	Up	tyrosinase, putative
MAC_05756	0.000000	1.443539	0.000000	Up	putative zinc finger protein odd-paired-like (opl)
MAC_05828	0.000000	1.577895	0.000000	Up	Cupin family protein
MAC_05875	0.000000	1.353232	0.000000	Up	serine family amino acid catabolism-related protein
MAC_05901	0.000000	1.058562	0.000000	Up	DUF1275 domain protein
MAC_06174	0.000000	1.101550	0.000000	Up	2-deoxyglucose-6-phosphate phosphatase
MAC_06221	0.000000	1.257796	0.000000	Up	WD-repeat containing protein slp1
MAC_06296	0.000000	1.998760	0.000000	Up	NlpC/P60-like cell-wall peptidase
MAC_06320	0.000000	1.112956	0.000000	Up	aspartic proteinase
MAC_06597	0.000000	1.485936	0.000000	Up	hypothetical protein
MAC_06620	0.000000	1.061755	0.000000	Up	zinc-binding dehydrogenase
MAC_06731	0.000000	-1.093226	0.000000	Down	ThiJ/PfpI family protein
MAC_06735	0.000000	-1.067103	0.000000	Down	arrestin
MAC_06779	0.000000	1.123749	0.000000	Up	NADPH-P450 reductase
MAC_07018	0.000000	-1.943428	0.000000	Down	extracellular serine-rich protein
MAC_07302	0.000000	4.444096	0.000000	Up	hypothetical protein
MAC_07323	0.000000	1.084462	0.000000	Up	ThiJ/PfpI family protein
MAC_07691	0.000000	1.136727	0.000000	Up	Hsp70 family chaperone
MAC_08480	0.000000	1.325977	0.000000	Up	copper amine oxidase 1

MAC_08916	0.000000	1.125432	0.000000	Up	cell surface protein
MAC_09371	0.000000	-1.260252	0.000000	Up	reverse transcriptase, putative
MAC_09485	0.000000	1.744783	0.000000	Up	hypothetical protein
MAC_09504	0.000000	1.377220	0.000000	Up	putative glucoamylase GMY2
MAC_00176	0.000000	0.000000	1.074034	Up	DUF895 domain membrane protein
MAC_01184	0.000000	0.000000	-1.651264	Down	ribose 5-phosphate isomerase
MAC_02150	0.000000	0.000000	1.268432	Up	peptidase family T4 protein
MAC_02174	0.000000	0.000000	1.087355	Up	mitochondrial cytochrome b2, putative
MAC_02548	0.000000	0.000000	1.297516	Up	hypothetical protein
MAC_02717	0.000000	0.000000	1.432964	Up	tyrosinase 2
MAC_02882	0.000000	0.000000	1.182703	Up	glycosyltransferase
MAC_03048	0.000000	0.000000	1.399847	Up	Fungal Zn binuclear cluster domain containing protein
MAC_04410	0.000000	0.000000	2.462847	Up	lysine amidinotransferase
MAC_04676	0.000000	0.000000	1.272493	Up	GNAT family acetyltransferase, putative
MAC_04833	0.000000	0.000000	1.191128	Up	hypothetical protein
MAC_05187	0.000000	0.000000	1.118570	Up	L-asparaginase, putative
MAC_05621	0.000000	0.000000	1.486234	Up	hypothetical protein
MAC_05709	0.000000	0.000000	1.930143	Up	uracil permease, putative
MAC_05902	0.000000	0.000000	1.044516	Up	carboxyphosphonoenolpyruvate phosphonomutase, putative
MAC_06568	0.000000	0.000000	2.366885	Up	integral membrane protein, putative
MAC_06676	0.000000	0.000000	-3.475582	Down	hypothetical protein
MAC_06850	0.000000	0.000000	1.081324	Up	cell wall protein
MAC_07250	0.000000	0.000000	1.333144	Up	cysteine synthase A
MAC_07741	0.000000	0.000000	1.323303	Up	putative cytochrome P450 3A7
MAC_08520	0.000000	0.000000	1.119107	Up	hypothetical protein
MAC_08796	0.000000	0.000000	1.755655	Up	exo-1,3-beta-D-glucanase
MAC_08826	0.000000	0.000000	1.148336	Up	serine/threonine-protein kinase Sgk2
MAC_09768	0.000000	0.000000	2.024539	Up	Amino-acid permease inda1

Table S3. 105 putative target genes of MaSep1.

Gene ID	Score	Predicted sequence
MAC_00171	10.83105	ATCAACAA
MAC_00187	9.870977	GTCAACAA
MAC_01539	10.88136	GTAAACAA
MAC_02330	9.492452	TTAAACAA
MAC_04376	9.775361	GAAAACAA
MAC_05784	9.775361	GAAAACAA
MAC_06623	9.203494	CAAAACAA
MAC_08195	9.203494	CAAAACAA
MAC_08514	9.328139	ATAAATAA
MAC_08656	9.299111	CTCAACAA
MAC_04635	10.73543	AAAAACAA
MAC_05216	9.775361	GAAAACAA
MAC_06624	9.203494	CAAAACAA
MAC_05447	10.73543	AAAAACAA
MAC_06937	9.775361	GAAAACAA
MAC_09524	9.725044	AACAACAA
MAC_09703	10.83105	ATCAACAA
MAC_00186	9.701628	ATAAACAG
MAC_00584	9.203494	CAAAACAA
MAC_00953	10.73543	AAAAACAA
MAC_01348	10.83105	ATCAACAA
MAC_04426	9.991104	ATAAACAT
MAC_05897	9.299111	CTCAACAA
MAC_06625	9.245241	ACAAACAA
MAC_06626	9.299111	CTCAACAA
MAC_07182	9.870977	GTCAACAA
MAC_07330	10.73543	AAAAACAA
MAC_07839	10.88136	GTAAACAA
MAC_08196	10.73543	AAAAACAA
MAC_08197	9.299111	CTCAACAA
MAC_08198	9.725044	AACAACAA
MAC_08199	11.84143	ATAAACAA
MAC_08200	11.84143	ATAAACAA
MAC_08201	9.492452	TTAAACAA
MAC_08795	9.299111	CTCAACAA
MAC_00623	9.725044	AACAACAA
MAC_01993	10.83105	ATCAACAA
MAC_03702	11.84143	ATAAACAA
MAC_03703	10.88136	GTAAACAA
MAC_05729	11.84143	ATAAACAA
MAC_05837	9.299111	CTCAACAA

MAC_07788	10.73543	AAAAACAA
MAC_08031	9.203494	CAAAACAA
MAC_08505	10.73543	AAAAACAA
MAC_00652	9.031036	GTAAACAT
MAC_00672	10.83105	ATCAACAA
MAC_01449	10.23209	ATAAACAC
MAC_02061	9.245241	ACAAACAA
MAC_02166	10.83105	ATCAACAA
MAC_02602	9.328139	ATAAATAA
MAC_02867	10.3095	CTAAACAA
MAC_03001	9.725044	AACAACAA
MAC_03043	9.775361	GAAAACAA
MAC_03050	9.203494	CAAAACAA
MAC_03761	9.221707	ATCAACAC
MAC_03899	9.725044	AACAACAA
MAC_04216	10.73543	ATCAACAA
MAC_04372	10.83105	ATCAACAA
MAC_04525	9.775361	GAAAACAA
MAC_04532	9.299111	CTCAACAA
MAC_04599	9.701628	ATAAACAG
MAC_04678	10.23209	ATAAACAC
MAC_04767	9.203494	CAAAACAA
MAC_04883	10.73543	AAAAACAA
MAC_04947	10.88136	GTAAACAA
MAC_05098	11.84143	ATAAACAA
MAC_05253	10.73543	AAAAACAA
MAC_05446	9.245241	ACAAACAA
MAC_05716	10.23209	ATAAACAC
MAC_05756	9.725044	AACAACAA
MAC_05875	9.870977	GTCAACAA
MAC_05901	10.83105	ATCAACAA
MAC_06296	9.203494	CAAAACAA
MAC_06320	10.73543	AAAAACAA
MAC_06597	10.73543	AAAAACAA
MAC_06620	9.725044	AACAACAA
MAC_06735	9.701628	ATAAACAG
MAC_06779	10.73543	AAAAACAA
MAC_07018	9.991104	ATAAACAT
MAC_07302	11.84143	ATAAACAA
MAC_07323	9.701628	ATAAACAG
MAC_07691	9.328139	ATAAATAA
MAC_08480	9.203494	CAAAACAA
MAC_09371	9.328139	ATAAATAA
MAC_09485	11.84143	ATAAACAA

MAC_00176	9.725044	AACAACAA
MAC_02150	10.23209	ATAAACAC
MAC_02174	10.3095	CTAAACAA
MAC_02548	10.73543	AAAAACAA
MAC_02717	9.870977	GTCAACAA
MAC_03048	10.88136	GTAAACAA
MAC_04410	10.23209	ATAAACAC
MAC_04676	9.870977	GTCAACAA
MAC_04833	9.245241	ACAAACAA
MAC_05621	9.299111	CTCAACAA
MAC_05902	10.83105	ATCAACAA
MAC_06568	9.775361	GAAAACAA
MAC_06676	9.328139	ATAAATAA
MAC_06850	9.031036	GTAAACAT
MAC_07250	9.328139	ATAAATAA
MAC_07741	9.870977	GTCAACAA
MAC_08520	9.725044	AACAACAA
MAC_08796	9.870977	GTCAACAA
MAC_08826	9.328139	ATAAATAA
MAC_09768	10.83105	ATCAACAA

Table S4. DEGs involved in conidiation pattern shift.

Gene ID	log2 (8 h)	log2 (10 h)	log2 (12 h)	Functions	References
MAC_06850	0.0000	0.0000	1.0813	Contributed to the integrity of the cell wall	[1]
MAC_04376	4.7628	3.6544	2.7971	Involved in conidial yield	[2]
MAC_07330	0.0000	3.3649	2.1688	Important for fungal growth and conidial yield	[2]
MAC_00623	1.1792	0.8334	0.0000	β-glucosidase is the rate-limiting enzyme for cellulase degradation	[3]
MAC_06625	0.0000	1.7427	0.0000	Involved in hypha morphology	[4]
MAC_08796	0.0000	0.0000	1.7557	Involved in hypha growth and conidial yield	[5]
MAC_06296	0.0000	1.9988	0.0000	Involved in cell cycle	[6]
MAC_04676	0.9276	0.9190	1.2725	Contribute to Chitin metabolism and cell wall rearrangement	[7]
MAC_09768	0.0000	0.0000	2.0245	Inhibition of glucose utilization	[7]
MAC_06568	0.0000	0.0000	2.3669	Contribute to cell division	[8]
MAC_06624	1.0946	2.0173	0.0000	Involved in responses to multiple stresses	[9]
MAC_08514	1.1730	1.1180	1.4092	Related to fungal resistance	[10]
MAC_00186	0.0000	4.0181	2.6722	Involved in hypha growth and conidiation pattern shift	[11]
MAC_03001	0.0000	1.8107	0.9082	Involved in hypha growth	[12]
MAC_08197	0.0000	2.6272	2.3918	Involved in the transport of	[13]

MAC_07250	0.8758	0.0000	1.3331	monocarboxylate contribute to hyphal growth	[14]
MAC_04410	0.0000	0.0000	2.4628	Involved in growth	[15]
MAC_01348	0.0000	1.5317	1.2413	Involved to cell cycle	[16]
MAC_04216	0.0000	-1.2044	0.0000	contribute to hyphal growth and virulence	[17]
MAC_04372	0.0000	1.2725	0.0000	Related to fungal resistance	[18]
MAC_01993	1.0979	0.0000	0.0000	Related to hyphal growth and virulence	[19]
MAC_06623	1.5867	0.0000	2.3647	Involved in growth	[20]
MAC_02061	0.0000	1.3119	0.0000	Important for fungal growth and fungal resistance	[21]
MAC_09524	2.4409	2.9087	2.4645	Contributed to the integrity of the cell wall	[22]
MAC_08199	0.0000	0.0000	2.4526	contribute to conidial yield	[23]

References

1. Free, S.J. Fungal cell wall organization and biosynthesis. *Adv Genet.* 2013, *81*, 33-82.10.1016/b978-0-12-407677-8.00002-6
2. Sevim, A., B.G. Donzelli, D. Wu, Z. Demirbag, D.M. Gibson and B.G. Turgeon. Hydrophobin genes of the entomopathogenic fungus, Metarhizium brunneum, are differentially expressed and corresponding mutants are decreased in virulence. *Curr Genet.* 2012, *58*, 79-92.10.1007/s00294-012-0366-6
3. Zhao, J., D. Shi, S. Yang, H. Lin and H. Chen. Identification of an intracellular β-glucosidase in *Aspergillus niger* with transglycosylation activity. *Appl Microbiol Biotechnol.* 2020, *104*, 8367-8380.10.1007/s00253-020-10840-4
4. Liu, L., Y. Yan, J. Huang, T. Hsiang, Y. Wei, Y. Li, J. Gao and L. Zheng. A Novel MFS Transporter Gene ChMfs1 Is Important for Hyphal Morphology, Conidiation, and Pathogenicity in Colletotrichum higginsianum. *Front Microbiol.* 2017, *8*, 1953.10.3389/fmicb.2017.01953
5. Tamano, K., Y. Satoh, T. Ishii, Y. Terabayashi, S. Ohtaki, M. Sano, T. Takahashi, Y. Koyama, O. Mizutani, K. Abe, et al. The beta-1,3-exoglucanase gene *exgA* (*exg1*) of *Aspergillus oryzae* is required to catabolize extracellular glucan, and is induced in growth on a solid surface. *Biosci Biotechnol Biochem.* 2007, *71*, 926-934.10.1271/bbb.60591
6. Hou, S., G. Chen, W. Wang, L. Xia, Z. Wang and Y. Lu. Identification of a cell-wall peptidase (NlpC/P60) from Nocardia seriolae which induces apoptosis in fathead minnow cells. *Journal of Fish Diseases.* 2020, *43*, 571-581.10.1111/jfd.13154

7. Lah, L., B. Podobnik, M. Novak, B. Korošec, S. Berne, M. Vogelsang, N. Kraševec, N. Zupanec, J. Stojan, J. Bohlmann, et al. The versatility of the fungal cytochrome P450 monooxygenase system is instrumental in xenobiotic detoxification. *Molecular Microbiology.* 2011, *81*, 1374-189.10.1111/j.1365-2958.2011.07772.x
8. Sethi, K., S. Palani, J.C. Cortés, M. Sato, M. Sevugan, M. Ramos, S. Vijaykumar, M. Osumi, N.I. Naqvi, J.C. Ribas, et al. A New Membrane Protein Sbg1 Links the Contractile Ring Apparatus and Septum Synthesis Machinery in Fission Yeast. *PLoS Genet.* 2016, *12*, e1006383.10.1371/journal.pgen.1006383
9. Tong, S.M., Y. Chen, S.H. Ying and M.G. Feng. Three DUF1996 proteins localize in vacuoles and function in fungal responses to multiple stresses and metal Ions. *Scientific Reports.* 2016, *6*, 20566-20576.10.1038/srep20566
10. Schmidt, F., A. Thywißen, M. Goldmann, C. Cunha, Z. Cseresnyés, H. Schmidt, M. Rafiq, S. Galiani, M.H. Gräler, G. Chamilos, et al. Flotillin-dependent membrane microdomains are required for functional phagolysosomes against fungal infections. *Cell Reports.* 2020, *32*, 108017-108039.10.1016/j.celrep.2020.108017
11. Bailey, L.A. and D.J. Ebbole. The fluffy gene of *Neurospora crassa* encodes a Gal4p-type C6 zinc cluster protein required for conidial development. *Genetics.* 1998, *148*, 1813-1820.10.1093/genetics/148.4.1813
12. Teichert, S., J.C. Rutherford, M. Wottawa, J. Heitman and B. Tudzynski. Impact of ammonium permeases mepA, mepB, and mepC on nitrogen-regulated secondary metabolism in *Fusarium fujikuroi*. *Eukaryot Cell.* 2008, *7*, 187-201.10.1128/ec.00351-07
13. Semighini, C.P., M.H. Goldman and G.H. Goldman. Multi-copy suppression of an *Aspergillus nidulans* mutant sensitive to camptothecin by a putative monocarboxylate transporter. *Current Microbiology.* 2004, *49*, 229-233.10.1007/s00284-004-4293-8
14. Fujita, Y. and K. Takegawa. Characterization of two genes encoding putative cysteine synthase required for cysteine biosynthesis in *Schizosaccharomyces pombe*. *Journal of the Agricultural Chemical Society of Japan.* 2004, *68*, 306-311.10.1271/bbb.68.306
15. YAN, Y. The screening and identification of A lysine 6-aminotransferase-producing fungi. *Pharmaceutical Biotechnology* 2007, *14*, 259-263.10.19526/j.cnki.1005-8915.2007.04.006
16. Iida, M., T. Taira, H. Ariga and S.M. Iguchi-Ariga. nduction of apoptosis in HeLa cells by MSSP, c-myc binding proteins. *Biological & Pharmaceutical Bulletin.* 1997, *20*, 10-15.10.1248/bpb.20.10
17. Liu, X., X. Pan, D. Chen, C. Yin, J. Peng, W. Shi, L. Qi, R. Wang, W. Zhao, Z. Zhang, et al. Prp19-associated splicing factor Cwf15 regulates fungal virulence and development in the rice blast fungus. *Environ Microbiol.* 2021, *23*, 5901-5916.10.1111/1462-2920.15616
18. Singh, P.K., A.K. Shrivastava, S. Singh, R. Rai, A. Chatterjee and L.C. Rai. Alr2954 of *Anabaena sp.* PCC 7120 with ADP-ribose pyrophosphatase activity bestows abiotic stress tolerance in *Escherichia coli*. *Functional & Integrative Genomics.* 2017, *17*, 39-52.10.1007/s10142-016-0531-y
19. Yao, S.H., Y. Guo, Y.Z. Wang, D. Zhang, L. Xu and W.H. Tang. A cytoplasmic Cu-Zn superoxide dismutase SOD1 contributes to hyphal growth and virulence of *Fusarium graminearum*. *Fungal Genetics and Biology.* 2016, *91*, 32-42.10.1016/j.fgb.2016.03.006
20. Mingot, J.M., M.A. Peñalva and J.M. Fernández-Cañón. Disruption of phacA, an *Aspergillus nidulans* gene encoding a novel cytochrome P450 monooxygenase catalyzing phenylacetate 2-hydroxylation, results in penicillin overproduction. *Journal of Biological Chemistry.* 1999, *274*, 14545-50.10.1074/jbc.274.21.14545

21. Ďurišová, K., M. Šimkovič, Ľ. Varečka, M. Kaliňák, K. Šoltys, B. Mosná and S. Kryštofová. Functional dissection of gamma-aminobutyric acid metabolism in *Neurospora crassa*. *General physiology and biophysics.* 2020, *39*, 205-218.10.4149/gpb_2020010
22. Wang, H., S. Sun, W. Ge, L. Zhao, B. Hou, K. Wang, Z. Lyu, L. Chen, S. Xu, J. Guo, et al. Horizontal gene transfer of *Fhb7* from fungus underlies Fusarium head blight resistance in wheat. *Science.* 2020, *368*, 1-7.10.1126/science.aba5435
23. Norvienyeku, J., Z. Zhong, L. Lin, X. Dang, M. Chen, X. Lin, H. Zhang, W.M. Anjago, L. Lin, W. Abdul, et al. Methylmalonate-semialdehyde dehydrogenase mediated metabolite homeostasis essentially regulate conidiation, polarized germination and pathogenesis in *Magnaporthe oryzae*. *Environmental Microbiology.* 2017, *19*, 4256-4277.10.1111/1462-2920.13888
